# Two-stage hybrid feature selection algorithms for diagnosing erythemato-squamous diseases

**DOI:** 10.1186/2047-2501-1-10

**Published:** 2013-05-30

**Authors:** Juanying Xie, Jinhu Lei, Weixin Xie, Yong Shi, Xiaohui Liu

**Affiliations:** School of computer science, Shaanxi Normal University, Xi’an, 710062 China; School of Information Engineering, Shenzhen University, Shenzhen, 518060 China; CAS Research Centre of Fictitious Economy & Data Science, Chinese Academy of Sciences, Beijing, 100080 China; School of Information systems, Computing and Mathematics, Brunel University, London, UB8 3PH UK

## Abstract

This paper proposes two-stage hybrid feature selection algorithms to build the stable and efficient diagnostic models where a new accuracy measure is introduced to assess the models. The two-stage hybrid algorithms adopt Support Vector Machines (SVM) as a classification tool, and the extended Sequential Forward Search (SFS), Sequential Forward Floating Search (SFFS), and Sequential Backward Floating Search (SBFS), respectively, as search strategies, and the generalized F-score (GF) to evaluate the importance of each feature. The new accuracy measure is used as the criterion to evaluated the performance of a temporary SVM to direct the feature selection algorithms. These hybrid methods combine the advantages of filters and wrappers to select the optimal feature subset from the original feature set to build the stable and efficient classifiers. To get the stable, statistical and optimal classifiers, we conduct 10-fold cross validation experiments in the first stage; then we merge the 10 selected feature subsets of the 10-cross validation experiments, respectively, as the new full feature set to do feature selection in the second stage for each algorithm. We repeat the each hybrid feature selection algorithm in the second stage on the one fold that has got the best result in the first stage. Experimental results show that our proposed two-stage hybrid feature selection algorithms can construct efficient diagnostic models which have got better accuracy than that built by the corresponding hybrid feature selection algorithms without the second stage feature selection procedures. Furthermore our methods have got better classification accuracy when compared with the available algorithms for diagnosing erythemato-squamous diseases.

## Introduction

The study of diagnosing erythemato-squamous diseases has become very popular since 1998 [1]. There are many experts including those in medicine and those in computer science, especially in artificial intelligence area, devote themselves to studying the diagnoses of erythemato-squamous diseases [2–11]. The erythemato-squamous diseases are very often seen in outpatient dermatology departments [1, 2]. There are six groups of the diseases, including psoriasis, seboreic dermatitis, lichen planus, pityriasis rosea, chronic dermatitis and pityriasis rubra pilaris. These six groups often share many clinical features of erythema with very few differences, so it is very difficult to perform a differential diagnosis for erythemato-squamous diseases in dermatology.

Furthermore, about the erythemato-squamous diseases, there are total 34 features, among which there are 12 clinical features obtained by a biopsy, and 22 histopathological features determined by an analysis of the skin samples under a microscope [1]. It is a common phenomenon that one disease may show features of another at the initial stage and display its own characteristic features at the following stages, which aggravate the difficulties for the differential diagnosis of erythemato-squamous disease according to its features, and attract more and more experts come from different areas focusing on the study of the diagnostic of erythemato-squamous diseases.

The available work about the diagnosing of erythemato-squamous diseases mainly involve using the different machine learning methods to uncover an efficient method to help doctors to make a right decision about the disease type based on its features. A common character of the current work is that they apply machine learning approaches directly to the problem without performing feature selection. However, feature selection for classification can preserve the key features to build an efficient classifier and remove the noisy and redundant ones, so that the classification rules are concise and the accuracy of classification becomes high. Therefor, in this paper we will do some related studies and try to discover an efficient feature selection method to build the sound diagnostic model to help doctors to make a right diagnostic decision.

In this connection, Liu *et al*[12] proposed a feature selection algorithm with dynamic mutual information, and adopted four typical classifiers to study the diagnoses of erythemato-squamous diseases. Karabatak and Ince [13] gave another feature selection method based on association rules and neural network. We suggested efficient feature selection methods to diagnose the diseases using the popular machine learning technique SVM (Support Vector Machines, SVM) as a classification tool [14, 15].

Although our previous feature selection methods gave strong results, they suffer the following two problems. One is the accuracy measure to evaluate the performance of a classifier may cause the skew problems. The other is the lack of the stability in the selected feature subsets. For example, if we face to a binary classification problem where there are 90 samples in the first group, and 10 in the second group. Under this condition, if all samples are classified into the first class and none into the second class, then the accuracy is 90%. However, this situation is the worst-case because the whole samples in the second class are misclassified. If it is the case where the 10 samples are the cancer patients and the other 90 are normal people, then even we have got 90% classification accuracy, but we cannot recognize any cancer patients from normal people. This situation is the one we should avoid. Therefore the traditional accuracy need correcting, so that it can reflect the performance of a classifier on each class. This is the motivation of the new accuracy being proposed in this paper. In addition, the selected feature subset may often not stable, especially when we did m-fold cross validation experiments to get the statistical and meaningful results, where the selected feature subset in each fold may not often same. This is the other motivation of this paper.

To overcome these disadvantages we propose the two-stage hybrid feature selection algorithms to diagnose the erythemato-squamous diseases, where we first present a new accuracy definition and use it to evaluate the performance of the corresponding temporary classifiers built on the related feature subsets in the feature selection procedures of our new hybrid feature selection algorithms. We carry out 10-fold cross validation experiments in the first stage and get 10 feature subsets, then in the second stage we merge the 10 selected feature subsets as the whole feature set and repeat our new hybrid feature selection algorithms on the partition which has got the best accuracy in the first stage, so that we can get the stable feature subset to build an efficient classifier for diagnosing erythemato-squamous diseases.

This paper is organized as follows. Section ‘Hybrid feature selection algorithms’ describes the principal feature selection method and the definition of the new accuracy and our new hybrid feature selection algorithms. Section ‘Experiments and analysis’ demonstrates our experimental results and analyzes them in detail. Finally, section ‘Conclusion’ draws conclusions and describes the future work.

## Hybrid feature selection algorithms

Feature selection plays an important role in building a classification system [16–19]. It can not only reduce the dimensionality of data, but also reduce the computational cost and gain a good classification performance.

The general feature selection algorithms comprise two categories: the filter and wrapper methods [20, 21]. The filter methods identify a feature subset from original feature set via a given evaluation criterion that is independent of learning algorithms. While the wrappers choose those features with high prediction performance estimated by a specific learning algorithm. The filters are efficient because of its independence of learning algorithms, while wrappers can obtain higher classification accuracy with the deficiency in generalization and computational cost. So there are more and more experts focus on studying the hybrid feature selection methods in recent decades for the hybrid feature selection methods can combine the advantages of filters and wrappers to uncover the classifiers with excellent performance.

This paper will present several two-stage hybrid feature selection algorithms. These algorithms take two steps to construct the stable and efficient classifiers. In the first step, the generalized F-score is adopted to rank features, and our extending SFS and SFFS and SBFS are used to select the necessary features to comprise the selected feature subset whist the performance of the temporary SVM evaluated with our modified accuracy is used to guide the feature selection procedure. 10-fold cross validation experiments have been conducted in the first stage. Then in the second step the 10 feature subsets selected in the first step are merged as a new original feature set, and the hybrid feature selection algorithms are executed again on the partition that has got the best performance among the 10 partitions in the first stage. The stable and efficient classifier will be built via training the exemplars in the subset of 9-fold and tested by the samples in the remaining 1-fold of the chosen partition.

**Figure 1:**
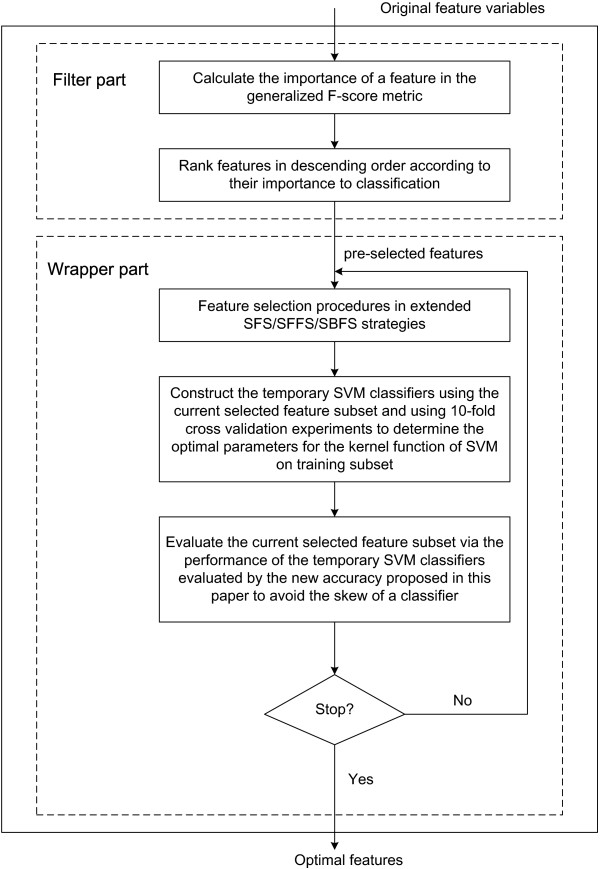
**New hybrid feature selection algorithms.**

Figure 1 illustrates the main idea of our hybrid feature selection algorithms. Where, the Generalized F-score is used to guide the application of filters, while the extended SFS/SFFS/SBFS with SVM combined our modified accuracy criterion are employed as wrappers. We rank features in descending order. The extended SFS and SFFS and SBFS is adopted to select the important or necessary features one by one by constructing many temporary SVM classifiers, whilst SVM with our new accuracy criterion is as a classification tool to direct the feature selection procedure.

Here we respectively introduce the generalized F-score and the definition of our new accuracy and our proposed three hybrid feature selection algorithms in the following subsections.

### Generalized F-score

The original F-score is to measure the discrimination of one feature between two sets of real numbers [18]. We generalized it in [14] to measure the discrimination of one feature between more than two sets of real numbers, so that it can value the importance of a feature to the classification in a multi-category classification problem. Here is the definition of the generalized F-score. Given training vectors *x*_*k*_, *k*=1,2,⋯,*m*, and the number of subsets *l*(*l*≥2), if the size of the *j*th subset is *n*_*j*_, *j*=1,2,⋯,*l*, then the F-score of the *i*th feature is *F*_*i*_. 1

where  and  are the average of the *i*th feature on the whole dataset and on the *j*th subset respectively, and  is the *i*th feature of the *k*th instance in the *j*th subset. The numerator of the right-hand side of equation (1) indicates the discrimination of the *i*th feature between each subset, and the denominator is the one within each subset. So the larger the *F*_*i*_ is, the more discriminative the *i*th feature is.

### The new classification accuracy measure

The accuracy of a classifier is often measured in the following equation (2). 2

where *N*_*r*_ is the number of samples which are classified correctly, and *N* is the total number of samples which are to be classified. This accuracy does not consider the performance of a classifier on each class, which may lead the skew of a classifier on some classes. For example, there is a cancer diagnostic problem with 90 normal people and 10 cancer patients. Now we have got a classifier that can recognize all normal people and zero cancer patients. Although the accuracy of the classifier is 90%, it is not a good one. So we define a new accuracy in the equation (3). 3

where *l* is the number of classes which are to be considered in a classification problem, and the  is the number of samples which are correctly classified in the *c*th class, and *N*^*c*^ is the total number of samples which are to be classified in the *c*th class. This new accuracy does consider the performance of a classifier on each class that is considering in the classification problem, so that the new accuracy can overcome the skew of a classifier when it is used to evaluate the performance of a classifier to guild the feature selection procedure.

### Several hybrid feature selection algorithms

Here are the related issues and our proposed hybrid feature selection algorithms which will comprise our two-stage hybrid feature selection algorithms for diagnosing erythemato-squamous diseases.

#### Feature search strategies

The traditional and still popular feature search strategies include sequential forward search (SFS) [22] and sequential backward search (SBS) [23] and sequential forward floating search (SFFS) and sequential backward floating search (SBFS) [24].

Here the aforementioned traditional SFS, SFFS, and SBFS strategies are extended as the followings. Firstly the features are ranked according to their F-score values, here the generalized F-score is used, then the features are dealt with one by one. In the extended SFS, features are selected according to their rank order, not as the traditional SFS which selects the feature that must be the best one when combined with the selected ones. And in the extended SFFS, we first trying to add a feature according to its rank order, then in the floating procedure we test the feature to be indeed added or not according to the new accuracy of the temporary classifier goes up or not, if the new accuracy of the temporary classifier goes up, then the related feature will be added to the selected feature subset, otherwise it will not be added. Similarly, in the extended SBFS, the procedure starts with all feature included, then at the following steps, the current lowest rank feature is tested deleting or not, if the accuracy of the temporary classifier without the feature becomes worse evaluated in our new accuracy, then the feature will not be deleted, otherwise it will be deleted. These procedures continue until all features are tested. The extended SFS and SFFS and SBFS are respectively faster than the traditional SFS and SFFS and SBFS in determining one feature to be selected or not in feature selection procedures.

#### Search the best parameters for SVM classifiers

SVM is the very popular and one of the best machine learning techniques, while some parameters must be provided to get the largest margin classifiers. So in order to get the optimal SVM classifier, the very simple and direct grid search technique is adopted here in 10-fold cross validation experiments on training subsets to discover the best parameter pairs (*C*,*γ*) for the RBF kernel function of SVM in our hybrid feature selection algorithms, so that we can get the separating hyperplane with the largest margin, i.e., the optimal classier. The range of *C* and *γ* we considering are *log*_2_*C*={−5,−4,−3,⋯,13,14,15} and *log*_2_*γ*={−15,−13,−11,⋯,3,1,3}, respectively.

#### Our hybrid feature selection algorithms

Here are the three hybrid feature selection algorithms, named new GFSFS, new GFSFFS and new GFSBFS, respectively. The generalized F-score plays the role of filters, and our extending SFS and SFFS and SBFS, respectively, with SVM and our new accuracy act as wrappers. Using the three new hybrid feature selection algorithms, new GFSFS, new GFSFFS and new GFSBFS, the necessary features are selected and the redundant ones are eliminated, so that a sound predictor to diagnose erythemato-squamous diseases is constructed. The detail procedures of our new GFSFS, new GFSFFS and new GFSBFS are, respectively, described as followings.

The new GFSFS uses extending SFS strategy to uncover the important features in building a classifier according to their F-score values, and uses SVM as a classification tool. The new accuracy is adopted to judge the performance of the temporary SVM classifiers to guide the feature selection procedure. The feature subset is composed of features with which the classifier on training subset has got the best diagnosing result. The pseudo code of our new GFSFS is here.

step 1: Determine the training and testing subsets of exemplars; Initialize the selected-feature-subset empty, and selecting-feature-subset with all features;

step 2: Computing the F-score value for each feature by using the equation (1) on the training subset, and sort features in descending order according to their F-score values;

step 3: Add the top feature in the selecting-feature-subset to the selected-feature-subset, and deleted it from the selecting-feature-subset as well;

step 4: Train the training subset with features in selected-feature-subset to construct the temporary optimal SVM classifier, the optimal parameters of SVM are determined via the aforementioned grid search technique and 10-fold cross validation experiments on the training subset;

step 5: Classify exemplars in the test subset and record the accuracy;

step 6: go to step 3, until the selecting-feature-subset becomes empty;

Although the new GFSFS can get a comparable good performance in diagnosing erythemato-squamous diseases, it may suffer the weakness of feature subset “nesting” that is the nature of SFS. That is, one feature will not be discarded once it has been selected and added to the selected-feature-subset.

The coming hybrid feature selection algorithm, new GFSFFS, will overcome this disadvantage of the new GFSFS by considering the correlation between features, so once the new accuracy of a temporary classifier on training subset doesn’t go up, then the selected feature will only be deleted from the selecting-feature-subset but will not be added to the selected-feature-subset. The details of the new GFSFFS are as the followings.

Step 1: Calculate the F-score value for each feature via the generalized F-score defined in equation (1) on the training subset of this fold, and rank features in descending order according to their F-score values; Initialize the selected-feature-subset empty and the selecting-feature-subset with all features;

Step 2: Delete the top feature from the selecting-feature-subset and add it to the selected-feature-subset;

Step 3: Train the training subset to build the optimal predictor model where the optimal parameter for the kernel function of SVM is determined in the aforementioned grid search technique and 10-fold cross-validation experiments on the training subset;

Step 4: If the new accuracy defined in equation (3) of the training subset is not improved, then the feature that has just been added will be eliminated from the selected-feature-subset;

Step 5: Go to Step 2 till all features in the selecting-feature-subset have been processed.

The features in the selected-feature-subset comprise the best feature subset of this fold, and the last SVM classifier is the optimal diagnostic model we are looking for on this fold.

To get a further self-contained demonstration of our new accuracy, we propose new GFSBFS hybrid feature selection algorithm and its procedure is here.

Step 1: Compute the F-score for each feature via the generalized F-score in equation (1) on this fold training subset, and rank features in descending order according to their F-score values; Initialize the selected-feature-subset with all features, and the visited tag for each feature unvisited;

Step 2: Train the training subset with features in the selected-feature-subset to build the optimal predictor model where the optimal parameter for the kernel function of SVM is determined by the aforementioned grid search technique and 10-fold cross validation experiments on the training subset; Record the accuracy of the model on training subset in the new accuracy defined in equation (3);

Step 3: Trying to delete the last unvisited feature in selected-feature-subset, and let the visited tag of it be visited;

Step 4: Train the training subset with features in the selected-feature-subset to build the optimal predictor model as step 2, and record the new accuracy of the model on training subset;

Step 5: If the new accuracy of training subset does not go up, keep the feature that it is trying to delete back to the selected-feature-subset, otherwise deleted it;

Step 6: Go to Step 3, until all features in the selected-feature-subset have been visited.

At last those features left in the selected-feature-subset are the necessary ones to build the optimal diagnostic model for this fold.

#### Two-stage hybrid feature selection algorithms

Because of the variation in the results of 10-fold cross validation experiments, we propose the two-stage hybrid feature selection algorithms. We do 10-fold cross validation experiments of our new GFSFS, new GFSFFS, and new GFSBFS in the first stage. Then we merge the 10 selected feature subsets of the 10-fold cross validation experiments as a new full feature set on which to carry out the following feature selection procedure of the second stage of our two-stage hybrid feature selection algorithms. In the second stage we repeat our new hybrid feature selection algorithms which are described in above subsection on the one partition which has got the best performance during the first stage, that is, the partition of the corresponding fold that has got the optimal accuracy in the 10-fold cross validation experiments in the pre-stage. In our experiment we choose the 10*th* fold, i.e., the last partition in the 10-fold cross validation experiments, to finish our two-stage hybrid feature selection algorithms.

## Experiments and analysis

This section first describes the erythemato-squamous diseases dataset from UCI machine learning repository [25], then demonstrates the experimental results we obtained on the dermatology dataset in detail, and analyzes the results in depth. This study is approved by Shaanxi Normal University, PR China.

### The erythemato-squamous diseases dataset

Table 1 is about the information of the erythemato-squamous diseases data set. It should be noted that we leave out 8 samples with missing values, so the samples in the data set used to do experiments in this paper is 358 not the original 366. In the dermatology dataset, that the value of the family history feature is 1 means one of these diseases has ever been observed in the family and 0 otherwise. The age feature is the patient age. Every other clinical or histopathological feature expresses the degree in the number from 0 to 3. Where, 0 means the feature is not present, 3 the largest amount possible, and 1, 2 the relative intermediate values.

**Table 1:** **Eryrhenato-squamous diseases dataset from UCI**

Diseases (patient number)	Clinical feature	Histopathological feature
Psoriasis (111)	Feature 1: Erythema	Feature 12: Melanin incontinence
Seboreic dermatitis (60)	Feature 2: Scaling	Feature 13: Eosrinophils in the infiltrate
Lichen planus (71)	Feature 3: Definite borders	Feature 14: PNL infiltrate
Pityriasis rosea (48)	Feature 4: Itching	Feature 15: Fibrosis of the papillary dermis
Chronic dermatitis (48)	Feature 5: Koebner phenomenon	Feature 16: Exocytosis
Pityriasis rubra pilaris (20)	Feature 6: Polygonal papules	Feature 17: Acanthosis
	Feature 7: Follicular papules	Feature 18: Hyperkeratosis
	Feature 8: Oral mucosal involvement	Feature 19: Parakeratosis
	Feature 9: Kneeand elbow involvement	Feature 20: Clubbing of the rete ridges
	Feature 10: Scalp involvement	Feature 21: Elongation of the rete ridges
	Feature 11: Family history	Feature 22: Thinning of the suprapapillary epidermis
	Feature 34: Age	Feature 23: Pongiform pustule
		Feature 24: Munro microabcess
		Feature 25: Focal hypergranulosis
		Feature 26: Disappearance of the granular layer
		Feature 27: Vacuolization and damage of basal layer
		Feature 28: Spongiosis
		Feature 29: Saw-tooth appearance of retes
		Feature 30: Follicular horn plug
		Feature 31: Perifollicular parakeratosis
		Feature 32: Inflammatory mononuclear infiltrate
		Feature 33: Band-like infiltrate

### Experimental results and analysis

Our aim is to construct an optimal diagnostic model to determine the types of erythemato-squamous diseases according to their features. In order to get a sound and statistical classifier we did 10-fold cross validation experiments on the datasets in the first stage. We repeat choosing the *i*th sample from each class to construct the *i*th fold, until each sample in the class is chosen, so that we averagely partition the whole dataset into 10 folds. We chose one fold as the testing subset, and the other nine folds as training subset. After that we use our new GFSFS, new GFSFFS and new GFSBFS, respectively, to construct the optimal diagnostic models. This procedure is iterated until each fold is chosen as a testing subset. At last we have got 10 feature subsets for each algorithm. These 10 selected feature subsets are merged together to establish the new full feature sets for each two-stage hybrid feature selection algorithm to carry out its second stage feature selection procedure.

We carry out experiments using the SVM library provided by Chang & Lin [26]. As a comparison we demonstrate the experimental results of the corresponding algorithms that use the original accuracy as the criterion to evaluate the temperary SVM classifiers to guild feature selection procedures. Tables 2 and 3 demonstrate the detail experimental results of 10-fold cross validation experiments of GFSFS and new GFSFS, respectively. Tables 4 and 5 show the results of 10-fold cross validation experiments of GFSFFS and new GFSFFS respectively. The 10-fold cross validation experimental results of GFSBFS and new GFSBFS are listed in Tables 6 and 7. Table 8 displays the new full feature sets for the two-stage hybrid feature selection algorithms to use in their second stage. Table 9 demonstrates the results of our two-stage hybrid feature selection algorithms. As a comparison, we list all the results of our hybrid feature selection algorithms including the results of one-stage and two-stage and those using traditional accuracy and new accuracy as different evaluation criterion to guild feature selection procedures respectively. It should be noted that the results of non two-stage hybrid feature selection algorithms in Table 9 is about the results on one partition where the second stage feature selection procedures are done for the two-stage hybrid feature selection algorithms. Table 10 summarizes the classification accuracies of all available methods on diagnosing erythemato-squamous diseases including this study. Where the first six results of this study is the average accuracy of 10-fold cross validation experiments of each hybrid feature selection algorithm in the first stage, and the next six is about the results of the corresponding two-stage hybrid feature selection algorithms.

**Table 2:** **Experimental results of GFSFS with ordinary accuracy**

Fold	Selected feature subset	Size of selected	Accuracy (%)
		feature subset	
1	33, 27,29, 31, 6, 12, 20, 15, 25, 22, 8, 7	22	100.0000
	21, 30, 9, 10, 16, 24, 28, 14, 5, 26		
2	33, 29, 27, 31, 6, 12, 15, 20, 25, 22, 7, 8	22	97.2222
	21, 30, 9, 24, 10, 28, 16, 14, 5, 26		
3	33, 27, 31, 29, 6, 12, 22, 25, 15, 20, 8, 7	23	100.0000
	30, 21, 9, 28, 10, 16, 24, 14, 5, 34, 26		
4	33, 27, 31, 29, 6, 12, 20, 15, 22, 7, 25, 8	23	94.4444
	21, 30, 9, 28, 16, 24, 10, 14, 5, 34, 26		
5	33, 27, 6, 31, 29, 12, 15, 22, 20, 25, 7, 8	21	100.0000
	21, 30, 9, 28, 24, 16, 10, 14, 5		
6	27, 33, 31, 6, 29, 12, 25, 22, 15, 20, 7, 8	23	100.0000
	21, 30, 9, 24, 16, 28, 10, 14, 5, 34, 26		
7	33, 27, 31, 29, 6, 12, 20, 15, 22, 7, 25, 8	21	100.0000
	21, 30, 9, 24, 28, 16, 10, 14, 5		
8	33, 27, 29, 12, 31, 6, 15, 22, 20, 7, 25, 8	23	97.2222
	21, 30, 9, 28, 16, 24, 10, 14, 5, 34,26		
9	33, 27, 29, 31, 6, 12, 22, 20, 25, 15, 7, 8	23	100.0000
	21, 30, 9, 28, 16, 24, 10, 14, 5, 34, 26		
10	33, 27, 31, 29, 6, 12, 20, 22, 15, 25, 8, 7	21	100.0000
	21, 30, 9, 28, 10, 24, 16, 14, 5		
Average & common	33, 27, 29, 31, 6, 12, 20, 15, 25, 22, 8, 7	22.20	98.89
	21, 30, 9, 10, 16, 24, 28, 14, 5

**Table 3:** **Experimental results of GFSFS with new accuracy**

Fold	Selected feature subset	Size of selected	Accuracy (%)
		feature subset	
1	33, 27, 29, 31, 6, 12, 20, 15, 25, 22, 8, 7	22	100.0000
	21, 30, 9, 10, 16, 24, 28, 14, 5, 26		
2	33, 29, 27, 31, 6, 12, 15, 20, 25, 22, 7, 8	22	97.2222
	21, 30, 9, 24, 10, 28, 16, 14, 5, 26		
3	33, 27, 31, 29, 6, 12, 22, 25, 15, 20, 8, 7, 30	23	100.0000
	21, 9, 28, 10, 16, 24, 14, 5, 34, 26		
4	33, 27, 31, 29, 6, 12, 20, 15, 22, 7, 25, 8	23	94.4444
	21, 30, 9, 28, 16, 24, 10, 14, 5, 34, 26		
5	33, 27, 6, 31, 29, 12, 15, 22, 20, 25, 7, 8	21	100.0000
	21, 30, 9, 28, 24, 16, 10, 14, 5		
6	27, 33, 31, 6, 29, 12, 25, 22, 15, 20, 7, 8	23	100.0000
	21, 30, 9, 24, 16, 28, 10, 14, 5, 34, 26		
7	33, 27, 31, 29, 6, 12, 20, 15, 22, 7, 25, 8	21	100.0000
	21, 30, 9, 24, 28, 16, 10, 14, 5		
8	33, 27, 29, 12, 31, 6, 15, 22, 20, 7, 25, 8	21	100.0000
	21, 30, 9, 28, 16, 24, 10, 14, 5		
9	33, 27, 29, 31, 6, 12, 22, 20, 25, 15, 7, 8	23	100.0000
	21, 30, 9, 28, 16, 24, 10, 14, 5, 34, 26		
10	33, 27, 31, 29, 6, 12, 20, 22, 15, 25, 8, 7	21	100.0000
	21, 30, 9, 28, 10, 24, 16, 14, 5		
Average & common	33, 27, 29, 31, 6, 12, 20, 15, 25, 22, 8, 7	22	99.17
	21, 30, 9, 10, 16, 24, 28, 14, 5

**Table 4:** **Experimental results of GFSFFS with ordinary accuracy**

Fold	Selected feature subset	Size of selected feature subset	Accuracy (%)
1	7, 31, 9, 5, 34, 4, 14, 28, 15, 17, 26, 25	12	100
2	5, 7, 14, 9, 31, 28, 15, 21, 16, 1, 17, 33, 18, 13	14	91.6667
3	26, 7, 31, 28, 9, 34, 15, 21, 14, 5, 2, 4, 17	13	100
4	7, 31, 30, 9, 5, 34, 28, 15, 21, 16, 4, 14, 1, 25, 33	15	88.8889
5	7, 31, 28, 15, 21, 5, 4, 14, 9, 34, 33, 29, 26, 18, 17	15	97.2222
6	7, 31, 9, 34, 28, 15, 21, 14, 16, 5, 33, 27, 26	13	97.2222
7	7, 31, 28, 9, 15, 21, 16, 14, 5, 4, 2, 33, 25	13	94.4444
8	7, 31, 33, 5, 28, 21, 15, 26, 29	9	97.2222
9	7, 31, 9, 34, 28, 21, 15, 5, 16, 14, 4, 2, 26, 17	14	94.1176
10	7, 31, 9, 28, 34, 15, 21, 5, 16, 4, 1, 18, 33, 32, 13	15	100
Average & common	7, 31, 5, 28, 15	13.3	96.08

**Table 5:** **Experimental results of GFSFFS with new accuracy**

Fold	Selected feature subset	Size of selected feature subset	Accuracy (%)
1	33, 29, 31, 6, 20, 15, 7, 21, 10, 16, 28, 14, 5, 26, 18	15	100.0000
2	33, 29, 31, 15, 20, 22, 7, 28, 16, 14, 5, 26, 18	13	94.4444
3	33, 31, 6, 22, 25, 15, 20, 7, 28, 10, 16, 14, 5, 26	14	100.0000
4	33, 31, 6, 20, 15, 7, 25, 21, 9, 28, 24, 14, 5, 26, 19	15	94.4444
5	33, 6, 31, 15, 22, 20, 25, 28, 16, 10, 14, 5, 4, 26	14	100.0000
6	27, 31, 22, 15, 20, 7, 16, 10, 14, 5, 26	11	97.2222
7	33, 31, 6, 20, 15, 22, 25, 28, 16, 10, 14, 5, 26, 18	14	100.0000
8	33, 29, 31, 6, 15, 22, 20, 16, 14, 5, 26	11	97.2222
9	33, 29, 31, 6, 22, 15, 9, 28, 16, 10, 14, 5, 26	13	100.0000
10	33, 31, 20, 15, 7, 21, 9, 28, 10, 14, 5, 26	12	100.0000
Average & common	31, 15, 14, 5, 26	13.2	98.33

**Table 6:** **Experimental results of GFSBFS with ordinary accuracy**

Fold	Selected feature subset	Size of selected feature subset	Accuracy (%)
1	17, 13, 19, 2, 3, 4, 34, 26, 5, 14, 28, 16	14	94.7368
	15, 33		
2	32, 18, 13, 17, 1, 26, 5, 14, 28, 15, 31, 29	12	94.4444
3	32, 18, 17, 13, 1, 19, 3, 23, 4, 26, 34, 5	18	100
	14, 16, 28, 9, 15, 33		
4	32, 13, 17, 1, 19, 2, 3, 23, 4, 26, 34, 5,14	19	97.2222
	9, 7, 22, 15, 31, 33		
5	1, 13, 17, 19, 2, 23, 26, 14, 24, 28, 9, 7	16	94.4444
	25, 22, 15, 33		
6	32, 18, 1, 13, 17, 19, 2, 3, 4, 26, 5, 28, 9	16	91.6667
	15, 31, 27		
7	32, 13, 18, 1, 17, 19, 2, 11, 4, 23, 3, 26	34	97.2222
	34, 5, 14, 10, 16, 28, 24, 9, 30, 21, 8, 25		
	7, 22, 15, 20, 12, 6, 29, 31, 27, 33		
8	32, 17, 13, 1, 3, 26, 5, 9, 30, 20, 22, 15	14	97.2222
	31, 33		
9	18, 13, 17, 1, 19, 2, 23, 3, 4, 26, 5, 14, 28	16	97.0588
	15, 31, 33		
10	32, 18, 13, 17, 1, 19, 11, 2, 3, 4, 23, 26	34	94.1176
	34, 5, 14, 16, 24, 10, 28, 9, 30, 21, 7, 8		
	25, 15,22, 20, 12, 6, 29, 31, 27, 33		
Average & common	17,13, 26, 15	19.3	95.81

**Table 7:** **Experimental results of GFSBFS with new accuracy**

Fold	Selected feature subset	Size of selected	Accuracy (%)
		feature subset	
1	13, 1, 19, 2, 4, 23, 34, 26, 5, 14, 28, 16, 7	15	97.36842105
	15, 33		
2	18, 13, 17, 19, 2, 3, 4, 23, 26, 5, 16, 28, 9	16	94.44444444
	22, 31, 33		
3	32, 18, 17, 13, 1, 19, 23, 4, 26, 34, 5, 14	18	94.44444444
	28, 9, 3 7, 15, 33		
4	32, 18, 13, 17, 19, 2, 3, 4, 26, 34, 5, 14	18	97.22222222
	16, 28, 7, 15, 20, 33		
5	1, 13, 17, 2, 3, 26, 4, 5, 28, 9, 7, 15, 33	13	94.44444444
6	18, 1, 13, 17, 2, 3, 4, 23, 26, 34, 5, 14, 28	18	91.66666667
	16, 15, 22, 31, 27		
7	13, 18, 1, 17, 23, 26, 34, 5, 14, 16, 7, 22	16	97.22222222
	15, 20, 27, 33		
8	32, 18, 17, 13, 1, 19, 2, 3, 4, 26, 34, 5, 10	21	88.88888889
	28, 9, 21, 25, 7, 22, 15, 27		
9	32, 13, 1, 11, 19, 2, 23, 3, 26, 34, 5, 14	21	97.05882353
	10, 24, 16, 28, 9, 21, 7, 15, 33		
10	13, 1, 19, 3, 4, 26, 5, 14, 28, 9, 15, 22, 29	15	100
	31, 33		
Average & common	13, 26, 5	17.1	95.28

**Table 8:** **The emerged feature subset for our two-stage hybrid feature selection algorithms**

For methods	Emerged feature subset of 10-fold cross validation experiment	Size of new full feature subset
Two-stage GFSFS	5, 6, 7, 8, 9, 10, 12, 14, 15, 16, 20, 21, 22, 24, 25, 26	23
	27, 28, 29, 30, 31, 33, 34	
Two-stage new GFSFS	5, 6, 7, 8, 9, 10, 12, 14, 15, 16, 20, 21, 22, 24, 25, 26	23
	27, 28, 29, 30, 31, 33, 34	
Two-stage GFSFFS	1, 2, 4, 5, 7, 9, 13, 14, 15, 16, 17, 18, 21, 25, 26, 27	23
	28, 29, 30, 31, 32, 33, 34	
Two-stage new GFSFFS	4, 5, 6, 7, 9, 10, 14, 15, 16, 18, 19, 20, 21, 22, 24, 25	22
	26, 27, 28, 29, 31, 33	
Two-stage GFSBFS	1, 2, 3, 4, 5, 6, 7, 8, 9, 10, 11, 12, 13, 14, 15, 16, 17	34
	18, 19, 20, 21, 22, 23, 24, 25, 26, 27, 28, 29, 30, 31	
	32, 33, 34	
Two-stage new GFSBFS	1, 2, 3, 4, 5, 7, 9, 10, 11, 13, 14, 15, 16, 17, 18, 19	30
	20, 21, 22, 23, 24, 25, 26, 27, 28, 29, 31, 32, 33, 34

**Table 9:** **Experimental results of our two-stage hybrid feature selection algorithms**

Methods	Selected feature subset	Size of selected feature subset	Accuracy (%)
GFSFS	33, 27, 31, 29, 6, 12, 20, 22, 15, 25, 8	21	100
	7, 21, 30, 9, 28, 10, 24, 16, 14, 5		
Two-stage GFSFS	22, 19, 17, 21, 2, 7, 11, 9, 3, 13, 15, 4	20	100
	12, 20, 5, 18, 14, 6, 10, 8		
New GFSFS	33, 27, 31, 29, 6, 12, 20, 22, 15, 25, 8	21	100
	7, 21, 30, 9, 28, 10, 24, 16, 14, 5		
Two-stage new GFSFS	22, 19, 17, 21, 2, 7, 11, 9, 3, 13, 15, 4	20	100
	12, 20, 5, 18, 14, 6, 10, 8		
GFSFFS	7, 31, 9, 28, 34, 15, 21, 5, 16, 4, 1, 18	15	100
	33, 32, 13		
Two-stage GFSFFS	22, 18, 20, 9, 13, 6, 17, 10, 8, 4, 15	11	100
New GFSFFS	33, 31, 20, 15, 7, 21, 9, 28, 10, 14, 5	12	100
	26		
Two-stage new GFSFFS	22, 20, 21, 12, 8, 4, 14, 16, 13, 5, 19, 6	16	100
	9, 7, 2, 17		
GFSBFS	32, 18, 13, 17, 1, 19, 11, 2, 3, 4, 23, 26	34	94.1176
	34, 5, 14, 16, 24, 10, 28, 9, 30, 21, 7, 8		
	25, 15,22, 20, 12, 6, 29, 31, 27, 33		
Two-stage GFSBFS	32, 13, 19, 2, 3, 26, 34, 5, 14, 16, 28, 9	15	100
	21, 15, 33		
New GFSBFS	13, 1, 19, 3, 4, 26, 5, 14, 28, 9, 15, 22	15	100
	29, 31, 33		
Two-stage new GFSBFS	28, 15, 10, 14, 1, 16, 2, 3, 20, 23, 30, 5	19	97.05882
	11, 13, 25, 7, 6, 12, 29

**Table 10:** **The accuracy comparison of all available classifiers**

Authors	Methods	Accuracy (%)
Übeyli and Güler (2005)	ANFIS	95.50
Luukka and Leppälampi (2006)	Fuzzy similarity-based classification	97.02
Polat and Günes (2006)	Fuzzy weighted pre-processing	88.18
	*K*-NN based weighted pre-processing	97.57
	Decision tree	99.00
Nanni (2006)	LSVM	97.22
	RS	97.22
	B1_5	97.50
	B1_10	98.10
	B1_15	97.22
	B2_5	97.50
	B2_10	97.80
	B2_15	98.30
Luukka (2007)	Similarity measure	97.80
Übeyli (2008)	Multiclass SVM with the ECOC	98.32
Polat and Günes (2009)	C4.5 and one-against-all	96.71
Übeyli (2009)	CNN	97.77
Liu et al. (2009)	Naïve Bayes	96.72
	1-NN	92.18
	C4.5	95.08
	PIPPER	92.20
Karabatak and Ince (2009)	AR and NN	98.61
Übeyli and Dog̈du (2010)	K-means clustering	94.22
Xie *et al* (2010)	IFSFFS	97.58
Xie *et al* (2011)	IFSFS	98.61
This study	GFSFS	98.89
	new GFSFS	99.17
	GFSFFS	96.08
	new GFSFFS	98.33
	GFSBFS	95.81
	new GFSBFS	95.28
	two-stage GFSFS	100
	two-stage new GFSFS	100
	two-stage GFSFFS	100
	two-stage new GFSFFS	100
	two-stage GFSBFS	100
	two-stage new GFSBFS	97.06

From the average accuracy of the 10-fold cross validation experiments listed in the last row of Tables 2 and 3 we can see that our new GFSFS outperforms GFSFS with the improvement in classification accuracy from 98.89% to 99.17%. The reason for the improvement in classification accuracy is that our new GFSFS adopted the new accuracy to value the performance of the temporary SVM in the feature selection procedure whilst the GFSFS used the traditional accuracy criterion to guild feature selection procedure. Tables 2 and 3 show that the selected features and test accuracy in each fold are nearly same except that in the fold eight. In this fold, our new GFSFS has got the 100% classification accuracy with 21 features, while the GFSFS only gets 97.22% accuracy and the selected feature subset is bigger than that of the new GFSFS with two more features. The average size of selected feature subset of our new GFSFS is smaller than the corresponding GFSFS. The common selected features of these two algorithms are same.

Tables 4 and 5 tell us that our new GFSFFS not only improves the classification accuracy of GFSFFS from 96.08% to 98.33%, but also reduces the dimension of dataset greatly. The size of selected feature subset is nearly the half of the corresponding GFSFS. The common selected features of new GFSFFS and GFSFFS are 5, 15, and 31, which is the subset of the common features of GFSFS and new GFSFS. These facts disclose the great efficiency of the new accuracy proposed in this paper in constructing the sound and efficient diagnostic models for diagnosing erythemato-squamous diseases.

Tables 6 and 7 show that the new GFSBFS algorithm cannot advance the accuracy of the GFSBFS diagnostic model except causing some extent reduction in dimension. The common features of these two GFSBFS are 13 and 26. Compared to the results displayed in Tables 2, 3, 4 and 5 of our other hybrid feature algorithms based on two different forward search strategies, we can say that the forward search strategy is better than the backward search strategy in finishing feature selection procedures to construct the sound and efficient diagnostic models for diagnosing erythemato-squamous diseases.

Feature 5 is the only one common feature of the new GFSFS and new GFSFFS and new GFSBFS algorithms, whilst feature 15 is the only one common feature of GFSFS and GFSFFS and GFSBFS. From this fact we can say that the feature 5 (Koebner phenomenon) and feature 15 (Fibrosis of the papillary dermis) are the most important features to be considered when establishing an efficient and sound diagnostic model. It can be noticed that feature 5 is a clinical feature and feature 15 is a histopathological feature. It demonstrate again that the differential diagnosing of erythemato-squamous diseases need consider both the clinical and histopathological features.

From Table 8 we can see that the new accuracy brings about the slightly smaller size of the full feature set for each hybrid feature selection algorithm to finish the feature selection procedure for the second stage.

It is clear in Table 9 that the two-stage GFSFS, two-stage new GFSFS, two stage GFSFFS, and two-stage GFSBFS outperform the GFSFS, new GFSFS, GFSFFS, and GFSBFS respectively. The two-stage new GFSFFS only keep the accuracy as the new GFSFFS, while with a slightly deficiency in the size of the selected feature subset. The two-stage new GFSBFS is outperformed by the new GFSBFS not only in the accuracy, but also in the dimension of the selected feature subset. From these analysis, we can say that the new accuracy definition improved the performance of the corresponding classifiers except for the two-stage GFSFFS and two-stage GFSBFS. So we can say that our new accuracy outperforms the traditional accuracy when used to guild the feature selection procedure to build a sound and efficient classifier, and our two-stage hybrid feature selection algorithms outperform the corresponding hybrid feature selection algorithms even the traditional accuracy is used to guild feature selection procedure. It seems that the new accuracy hasn’t brought any improvements in the two-stage hybrid feature selection algorithms when the forward or backward floating search strategy is used.

The summary in Table 10 demonstrates that among our hybrid feature selection algorithms the new GFSFS obtains the highest average accuracy of 99.17% to diagnose erythemato-squamous diseases, and our GFSFS follows, then is our new GFSFFS, GFSFFS, GFSBFS, and our new GFSBFS, respectively. It is clear that our new accuracy advances the performance of our hybrid feature selection algorithms except for that of the GFSBFS.

It can also be seen in the Table 10 that the two-stage hybrid feature selection algorithms can get 100% accuracy in diagnosing erythemato-squamous diseases except the two-stage new GFSBFS algorithm which has got 97.06% accuracy. The reasons for this good performance should be due to two aspects. One is that we conduct the two-stage hybrid feature selection algorithms; the other is that the second stage of our hybrid feature selection algorithms are executed on the one partition that has got the best results in the 10-cross validation experiments in the first stage.

From the above analysis it is clear that we have detected the diagnostic model that is better than the counterparts for diagnosing erythemato-squamous diseases by using our two-stage hybrid feature selection algorithms.

## Conclusion

A new accuracy definition was proposed in this paper, and it was used to evaluate the performance of a classifier to avoid the skew of it and to establish the sound diagnostic models for diagnosing erythemato-squamous diseases.

Several hybrid feature selection algorithms were proposed based on the generalized F-score and SVM with the new accuracy to value the performance of the temporary SVM to guide the feature selection procedures. The new hybrid feature selection algorithms combined the strengths of filters and wrappers to uncover the optimal feature subset with the best diagnostic efficiency and avoided the skew of a classifier as well.

The two-stage hybrid feature selection algorithms were proposed based on the new hybrid feature selection algorithms to construct the stable and efficient diagnostic models for erythemato-squamous diseases.

The experimental results show that our two-stage feature selection algorithms outperformed our hybrid feature selection algorithms and other available ones. They can construct effective diagnostic models that may help doctors to make a sound decision in diagnosing erythemato-squamous diseases. However, in order to get the models with the largest margin, we performed 10-fold cross validation experiments and grid search techniques for the optimal parameters of SVM on the training subsets, which incurred extra computation costs. Minimizing this cost is one of the directions for our future work.
